# Ceruloplasmin as a prognostic marker in patients with bile duct cancer

**DOI:** 10.18632/oncotarget.15995

**Published:** 2017-03-07

**Authors:** In Woong Han, Jin-Young Jang, Wooil Kwon, Taesung Park, Yongkang Kim, Kyoung Bun Lee, Sun-Whe Kim

**Affiliations:** ^1^ Department of Surgery, Samsung Medical Center, Sungkyunkwan University School of Medicine, Gangnam-Gu, Seoul 06351, Korea; ^2^ Department of Surgery and Cancer Research Institute, Seoul National University College of Medicine, Chongno-Gu, Seoul 110-744, Korea; ^3^ Department of Statistics, Seoul National University College of Natural Sciences, Gwanak-Gu, Seoul 08826, Korea; ^4^ Department of Pathology, Seoul National University College of Medicine, Chongno-Gu, Seoul 110-744, Korea

**Keywords:** bile duct, cancer, cholangiocarcinoma, biomarker, ceruloplasmin

## Abstract

**Background and Aims:**

Bile duct cancer is one of the lethal cancers, presenting difficulties in early diagnosis and limited treatment modalities. Despite current advances in biomarker research, most studies have been performed in Western populations. Therefore, the purpose of this study was to determine a prognostic marker for bile duct cancer, especially in Korean patients, whose incidence of bile duct cancer is high.

**Results:**

Comparing cancer and normal bile duct tissue, we identified 29091 differentially expressed genes. CP, SCEL, and MUC16 had positive coefficients with a log2 ratio >1 for advanced T, N stage and perineural invasion cancer tissue. Strong immunohistochemical expression of ceruloplasmin was dominant in tumors with advanced T stage (p>0.999) and perineural invasion (p=0.316).

**Patients and Methods:**

We performed tissue microarray experiment with 79 bile duct cancer tissue samples and 21 normal bile duct tissue samples. Candidate genes that has positive correlation with T, N stage and perineural invasion were drawn with multivariate analysis. Tissue expression of the genes was evaluated with an immunohistochemical study.

**Conclusions:**

Ceruloplasmin is supposed to be related with advanced T stage and perineural invasion, having a possibility as a candidate prognostic marker for bile duct cancer.

## INTRODUCTION

Biliary tract cancer is the 10^th^ most common cancer in the United States, and its mortality ranks 5^th^ among all cancers [1]. The incidence of biliary tract cancer is higher in Eastern than Western populations, and it is the 9^th^ most common cancer in Korea [2]. The actual 5-year survival rate for bile duct cancer after curative resection ranges from 28 to 30.1% [3, 4]. Radical resection of the tumor with a gross and microscopic negative resection margin is essential for long-term survival. However, surgical candidates are few because of difficulties in early diagnosis caused by asymptomatic manifestation, a lack of sensitive biomarkers, and the cancer's aggressiveness. In addition, treatment modalities for bile duct cancer are limited, because the cancer is refractory to chemotherapy and radiation treatment. Therefore, discovery of diagnostic, therapeutic, and prognostic biomarkers for biliary tract cancer is important.

Recently, there have been marked advances in biomarkers for biliary tract cancers [5]. Diagnostic markers as p38 [6], MMP [7], and miR-21 [8], therapeutic markers such as Erb-1/EGFR [9], VEGF [10], ERKs [11], PI3K [12], mTOR [13], and SMAD4 [14], and prognostic markers such as Erb-B3/Her3 [15], PTEN [16], CA19-9 [17], SMAD4 [18], IDH [19], miR-26a [20], and miR-192 [21] have all been suggested. However, the number of study subjects was relatively small, and some studies used *in vivo* experiments. Moreover, most of those studies were performed with a Western population. Therefore the purpose of this study was to determine prognostic markers for bile duct cancer, especially in Korean patients. To our knowledge, this study is the first conducted in Eastern bile duct cancer patients with a large number of study subjects.

## RESULTS

### Clinicopathological characteristics of the study subjects

The clinicopathological characteristics of the 79 bile duct cancer patients are listed in Table 1. The mean age of the study subjects was 65.4 years and the male to female ratio was 1.82 to 1. Jaundice was identified in 32 (40.5%) patients. Sixty-three patients (79.7%) had extrahepatic bile duct cancer, and curative resection was performed in 73 patients (92.4%). Tumors were confined to the bile duct in 14 patients (17.7%), lymph node metastasis was identified in 31 patients (39.2%), and perineural invasion was seen in 56 (70.9%) patients. The median follow-up with the patients was 33.3 months. Overall 5-year survival rate of all patients was 45.5%, and 57.2% after R0 resection. After R0 resection, 5-year survival rate was 78.6% for those with tumors limited to bile duct, and 51.8% for those with tumors extending beyond bile duct (p=0.067). Node negative patients had higher 5-year survival rate compared with node positive patients (70.1% vs. 0%, p=0.001). Perineural invasion negative patients had higher 5-year survival rate compared with perineural invasion positive patients (85.0% vs. 43.4%, p=0.004).

**Table 1:** Demographics and pathologic data

Variables	N=79
Age (years, mean ± SD)	65.4±7.7
Gender (male: female)	51:28
Location (Extrahepatic: Intrahepatic)	63 (79.7%): 16 (20.3%)
Location of extrahepatic bile duct cancer (Proximal: mid- to- distal)	28 (44.4%): 35 (55.6%)
Operative methods (Hepatectomy with BDR: BDR: PD)	30 (38.0%): 19 (24.1%): 30 (38.0%)
Curative resection	73 (92.4%)
Recurrence	37 (50.7%)(of 73 curative resection)
Follow-up duration (months, median, range)	33.3 (0.8-76.2)
Gross type (papillary: nodular: flat)	14 (17.7%): 41 (51.9%): 24 (30.4%)
Histologic grade (WD: MD: PD)	12 (15.2%): 51 (64.6%): 10 (12.7%)(of 73 patients)
Depth of invasion (confined to bile duct: beyond bile duct)	14 (17.7%): 65 (82.3%)
Lymph node metastasis	31 (39.2%)(of 71 patients)
Perineural invasion	56 (70.9%)

Abbreviations: BDR: bile duct resection, PD: pancreaticoduodenectomy, WD: well-differentiated, MD: moderate- differentiated, PD: poorly-differentiated.

### Differentially expressed gene analysis

Comparing cancer and normal bile duct tissue, 29091 differentially expressed gene (DEGs) were identified with adjusted p value (FDR correction) <0.05. According to T stage, we found 304 significant DEGs, of which 113 genes had a log2 ratio >0. The top 50 genes are listed in Supplementary Table 1. According to lymph node metastasis, we found no significant DEG with an adjusted p value <0.05. 2604 DEGs were identified with a p value <0.05, and 1262 genes had a positive coefficient (Supplementary Table 2). According to perineural invasion, 7 significant DEGs were identified and 5 of them had a positive coefficient (Supplementary Table 3).

### Candidate genes associated with advanced bile duct cancer

**Figure 1:**
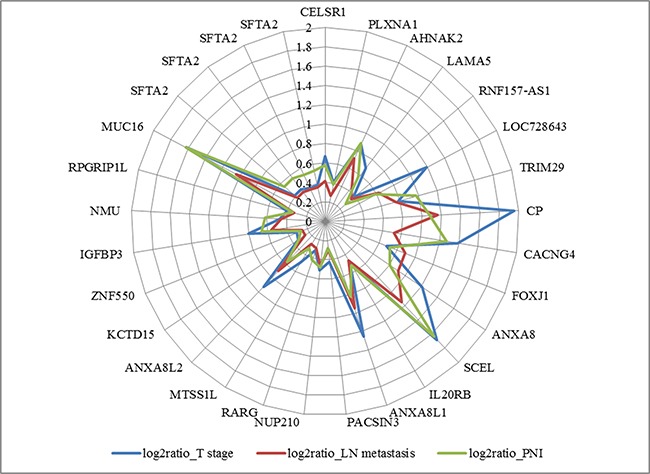
Diagram of positive coefficient of 29 genes with cancer to normal bile duct log2ratio >0, adjusted p-value <0.05

To find genes positively associated with increased T, N stage and positive perineural invasion, we performed multivariate analysis. Because we found no significant DEG for lymph node metastasis when applying adjusted p-value criteria, we included 157 genes with unadjusted p-value <0.05 in both T, N stage and perineural invasion in this analysis. The top 50 genes are listed in Table 2. Among the 477 genes we included in this analysis, we identified 199 significant DEGs, of which 29 had a positive coefficient with cancer tissue, advanced T, N stage, and perineural invasion (Figure 1).

**Table 2:** Top 50 genes with positive coefficient toward advanced T stage, N stage, and perineural invasion

log2. ratio ofcancer	T stagep-value	log2ratioof T stage	N stagep-value	log2ratio ofN stage	PNIp-value	log2ratio ofPNI	Multipleregressionp-value	Adjustedp-value	Gene symbol
1.405649235	0.005021	0.669221179	0.049282	0.414807777	0.0066929	0.577734589	8.67E-12	1.85E-09	CELSR1
0.902487966	0.017299	0.408004864	0.048754	0.274492969	0.0062699	0.388747711	8.01E-10	5.33E-08	PLXNA1
1.336755747	0.005524	0.878416145	0.010444	0.71439579	0.0018927	0.883976089	6.28E-08	1.46E-06	AHNAK2
0.774103937	0.000954	0.687681207	0.008804	0.45790676	0.0019398	0.569426772	5.74E-06	4.81E-05	LAMA5
0.568306702	0.015232	0.381576062	0.006886	0.351554572	0.0458663	0.278862871	7.42E-06	5.92E-05	RNF157-AS1
1.230802084	0.000229	1.183765995	0.005831	0.630328305	0.0271156	0.588247469	1.04E-05	7.72E-05	LOC728643
1.168560622	0.0305	0.781214914	0.00944	0.751484198	0.0016675	0.976805933	2.13E-05	0.000136725	TRIM29
1.593130487	0.042005	1.075298616	0.044852	0.915541975	0.0030054	1.328602148	4.05E-05	0.000229513	---
1.755681238	0.000359	1.952839198	0.006969	1.159846203	0.0261894	1.083681984	4.41E-05	0.000246188	CP
1.256035134	0.000343	1.382233559	0.035821	0.723628435	0.0001428	1.271430559	4.65E-05	0.000256884	CACNG4
1.035723885	0.038296	0.680393235	0.000894	0.891679109	0.0103758	0.719665669	6.05E-05	0.000319024	FOXJ1
1.055140967	0.000506	1.21563373	0.000779	0.910845838	0.00771	0.80855569	0.000163	0.000718317	ANXA8
0.38719036	0.003896	0.381130034	0.046294	0.235723632	0.0367647	0.267271458	0.000187	0.000805512	---
1.422747031	0.001163	1.680970054	0.011585	1.149656434	0.0004231	1.636415885	0.000259	0.001056886	SCEL
0.741694744	0.041854	0.500599655	0.017604	0.472999653	0.0090105	0.526894352	0.000303	0.001201835	IL20RB
1.048336388	0.000656	1.256426426	0.00088	0.948583195	0.0102444	0.816245506	0.000367	0.001407568	ANXA8L1
0.439815207	0.007863	0.420094066	0.015973	0.298972856	0.0468603	0.276542029	0.000405	0.001524813	PACSIN3
0.644644964	0.031368	0.510770319	0.011303	0.457911463	0.0238407	0.477635525	0.000432	0.001607237	NUP210
0.41460036	0.03709	0.311652312	0.016663	0.291643291	0.000777	0.41712347	0.000651	0.002251733	RARG
0.459884319	0.004722	0.493286574	0.045051	0.27407048	0.0322201	0.323656785	0.000967	0.003135081	MTSS1L
0.752843756	0.001219	0.926599211	0.00119	0.702989327	0.0191047	0.575784542	0.000974	0.003154055	ANXA8L2
0.326934803	0.006896	0.339485505	0.023013	0.24777875	0.0025399	0.325922534	0.001317	0.004059408	KCTD15
0.327674175	0.021866	0.313482341	0.019959	0.25034948	0.036247	0.266040443	0.001513	0.004559996	ZNF550
0.638509872	0.01561	0.628376088	0.027736	0.496854056	0.0167853	0.527506439	0.001932	0.005596862	03-Sep
0.641215401	0.002851	0.801735895	0.018654	0.562292769	0.0076319	0.66523785	0.002029	0.005824709	IGFBP3
0.499649263	0.042891	0.470272503	0.016633	0.459860135	0.0013724	0.619993877	0.005756	0.014050979	NMU
0.401188962	0.03306	0.405520149	0.026384	0.332817538	0.0409198	0.34779977	0.00705	0.016707122	RPGRIP1L
1.038620022	0.001477	1.622430348	0.01432	1.04114733	0.0001852	1.62939924	0.009159	0.020847277	MUC16
0.565775908	0.01136	0.736079957	0.019033	0.5676243	0.0199131	0.594558424	0.012178	0.026551104	---
0.375493033	0.042879	0.410540932	0.031223	0.373539325	0.001184	0.554925038	0.017506	0.036142137	SFTA2
0.375493033	0.042879	0.410540932	0.031223	0.373539325	0.001184	0.554925038	0.017506	0.036142137	SFTA2
0.187599817	0.024383	0.226129552	0.024041	0.187735793	0.0367099	0.179514001	0.019273	0.039199952	---
0.344763407	0.048564	0.379079767	0.027571	0.359760333	0.000962	0.530485462	0.021143	0.042354375	SFTA2
0.344763407	0.048564	0.379079767	0.027571	0.359760333	0.000962	0.530485462	0.021143	0.042354375	SFTA2
0.276170322	0.005814	0.433065399	0.008952	0.325178256	0.02379	0.303523881	0.02891	0.055235829	OBSL1
0.311893948	0.016383	0.456822732	0.02489	0.351506827	6.82E-05	0.62551085	0.029778	0.056617243	DUSP7
0.29107782	7.23E-05	0.680455719	0.031919	0.356012678	0.0002128	0.580257197	0.034151	0.063494817	SLC25A12
0.763158464	0.021464	1.089699767	0.000956	1.272277802	0.0286497	0.863378185	0.034747	0.064421874	CYP24A1
0.463353083	0.000208	1.071169232	0.002294	0.763081908	0.0003075	0.938569956	0.037331	0.068426105	PLAG1
0.235225211	0.006514	0.412864124	0.004589	0.359195411	0.0184164	0.333983417	0.037393	0.068520091	TBL1X
0.415837064	0.020375	0.618452648	0.003429	0.642991749	0.0031283	0.661698671	0.038076	0.069551285	TP63
0.36489978	3.79E-05	0.952535143	0.037331	0.445880795	0.000191	0.781545771	0.04198	0.075557211	PTPRU
0.221020043	0.001706	0.490176098	0.007407	0.367678885	0.0031479	0.422636582	0.064293	0.108164516	TMEM194B
0.211173621	4.11E-05	0.598084608	0.007149	0.316272465	0.0025179	0.417098103	0.067198	0.112310859	ZNF772
0.244788935	0.007511	0.460172837	0.049158	0.291772733	0.0001663	0.540728636	0.068464	0.114089313	CYP27C1
0.196298462	0.046793	0.290292197	0.034977	0.247751009	0.0459685	0.264254519	0.073753	0.121551209	ZNF211
0.189809559	2.48E-06	0.65798624	0.037054	0.257415442	0.000286	0.467404599	0.092093	0.146412737	SRGAP2B
0.46529506	0.013098	0.919444033	0.001183	0.98387549	0.0054371	0.850979956	0.10161	0.159071083	DSC3
0.336275552	0.048061	0.513851773	0.02283	0.482223223	0.0287236	0.459517938	0.111838	0.172326235	KRT4
0.432728625	0.043834	0.730488208	0.015057	0.717167303	0.0035367	0.883170907	0.116773	0.178647079	TRPV4

A search of the PANTHER database showed that 23 of those genes were associated with molecular functions, biological processes, cellular components, protein classes, and pathways. Ten of those genes (43.5%) had a binding molecular function and 6 (26.1%) had receptor activity. According to biological process, 10 genes (43.5%) were involved in cellular processes, 9 (39.1%) in metabolic processes, and 7 (30.4%) in biological regulation. Among them, we identified top 3 candidate genes with a log2 ratio >1 for advanced T, N stage and perineural invasion (Table 3).

**Table 3:** Top 3 genes with positive coefficient >1, toward advanced T stage, N stage, and perineural invasion after multiple linear regression

log2-ratio ofcancer	T stagep-value	log2-ratioof T stage	N stagep-value	log2-ratioof N stage	PNIp-value	log2-ratio ofPNI	Multipleegressionp-value	Adjustedp-value	Gene symbol
1.755681	0.000359	1.952839	0.006969	1.159846	0.026189	1.083682	4.41E-05	0.000246	CP
1.422747	0.001163	1.68097	0.011585	1.149656	0.000423	1.636416	0.000259	0.001057	SCEL
1.03862	0.001477	1.62243	0.01432	1.041147	0.000185	1.629399	0.009159	0.020847	MUC16

### Immunohistochemical analysis of expression

**Figure 2:**
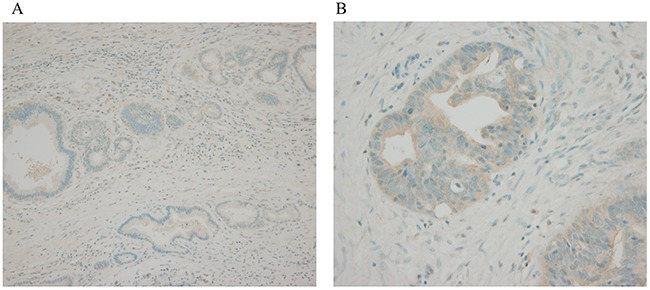
Immunohistochemical examination of bile duct cancer tissue **(A)** normal bile duct, no expression, x100 **(B)** tumor tissue, strong expression, x400

**Figure 3:**
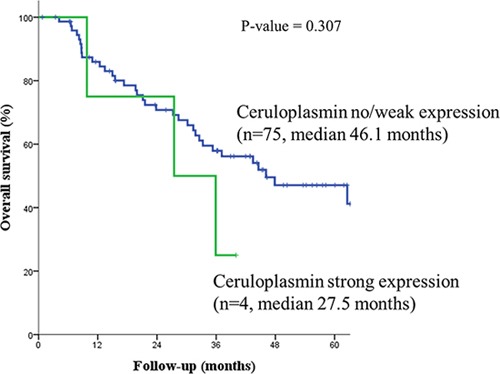
Patients with strong ceruloplasmin expression had shorter median overall survival than those with no or weak overexpression

Immunohistochemical staining of tumor samples for candidate genes associated with ceruloplasmin was weakly positive in 16 samples (20.3%), and strongly positive in 4 (5.1%, Figure 2). Ceruloplasmin was overexpressed in patients with jaundice (n=4, 75.0% vs. 38.7%, p=0.298), extrahepatic bile duct cancer (n=4, 6.3% vs. 0, p=0.577), tumors invading beyond the bile duct (n=4, 6.2% vs. 0, p>0.999), and perineural invasion (n=4, 7.1% vs. 0, p=0.316, Table 4). Patients with strong ceruloplasmin expression tended to have shorter median overall survival than those with no or weak overexpression (median survival 27.5 vs. 46.1 months, p=0.307, Figure 3).

**Table 4:** Clinical characteristics according to ceruloplasmin expression

	No or weak ceruloplasminexpression (n=75)	Strong ceruloplasminexpression(n=4)	p-value
Tumor location			0577
Intrahepatic bile duct	59 (78.7%)	4 (100%)	
Extrahepatic bile duct	16 (21.3%)	0	
T stage			>0.999
Confined to bile duct	14 (18.7%)	0	
Beyond bile duct	61 (81.3%)	4 (100%)	
N stage			0.627
N (−)	37 (55.2%)	3 (75.0%)	
N (+)	30 (44.8%)	1 (25.0%)	
Perineural invasion			0.316
Negative	23 (30.7%)	0	
Positive	52 (69.3%)	4 (100%)	
Curative resection			>0.999
Yes	69 (92.0%)	4 (100%)	
No	6 (8.0%)	0	

## DISCUSSION

Depth of tumor invasion into the bile duct, lymph node metastasis, perineural invasion, histologic differentiation, resection margin status, and tumor markers are all well-recognized prognostic factors for bile duct cancer [4]. However, predicting patient prognosis using those factors is inadequate, more effective prognostic biomarkers are needed. In this study, we drew a prognostic marker for bile duct cancer in Korean patients using microarray experiment with a robust statistical method. We used a statistical analysis to find genes positively associated with 3 well-recognized prognostic factors, T, N stage and perineural invasion. As a result, we found 3 novel candidate genes (CP, SCEL, and MUC16), having positive coefficients with a log2 ratio >1 for advanced T, N stage and perineural invasion cancer tissue. The authors selected ceruloplasmin for tissue expression analysis. It showed a positive correlation with advanced disease and poor prognosis.

Ceruloplasmin is a multicopper oxidase with functions including copper transport, ferroxidase activity, superoxide dismutase activity, and amine oxidase activity [22]. A low level of serum ceruloplasmin indicates Wilson disease [23], or aceruloplasminemia [24] and a high level of serum ceruloplasmin is related to copper toxicity, oral contraceptive pill use [25], inflammatory diseases [22], angina [26], Alzheimer's disease [27], schizophrenia [28], and obsessive-compulsive disorder [29].

Ceruloplasmin has also been reported to be related to several types of cancers. Elevated glycoconjugates could be the result of an inflammatory reaction associated with neoplasia, because serum ceruloplasmin which is an acute phase reactant, is also increased in those patients [30]. Ceruloplasmin was suggested as a promising marker for the patients with pancreatic ductal adenocarcinoma because it was highly secreted by PNAC1 cancer stem-like cells [31], especially those negative for CA19-9. [32] Inhibition of ceruloplasmin has been demonstrated to suppress tumor growth and angiogenesis in breast cancer [33]. In breast cancer, elevated ceruloplasmin has been found in patients with metastatic disease. In those patients, the ceruloplasmin level fell in response to treatment, and those with elevated post-mastectomy ceruloplasmin levels had a higher rate of recurrence [34]. Ceruloplasmin was also suggested to be a potentially reliable biomarker for the detection of hepatocellular carcinoma [35], especially in Hepatitis C virus-infected alcoholic patients [36]. A rise in serum ceruloplasmin was observed in cervical cancer, and that rise was higher in later stages of cancer than in early stages [30, 37]. Serum ceruloplasmin could complement biochemical screening in prostate carcinoma, [38] especially in cases without elevated serum PSA. [39] Ceruloplasmin levels were significantly increased in ovarian cancer patients compared with controls. [40] The ceruloplasmin promoter demonstrated significantly higher activities in ovarian cancers compared with normal organs [41], especially in patients with intrinsic chemoresistance [42]. Ceruloplasmin produced higher signals in the ascites fluids of epithelial ovarian cancer patients [43]. Ceruloplasmin was also suggested as a plasma biomarker of hypopharyngeal squamous cell carcinoma [44]. In this study, we found that ceruloplasmin was overexpressed 3.38 fold in tumor tissue compared with normal bile duct tissue. Moreover, strong expression of ceruloplasmin was observed in tumors with advanced T stage and perineural invasion.

On the other hand, a relation has been reported between bile duct obstruction and ceruloplasmin. In a rat model, common bile duct ligation brought about a rapid increase in serum ceruloplasmin concentration. [45] Primary biliary cirrhosis patients showed increased ceruloplasmin activity in the serum [46]. In this study, we observed strong expression of ceruloplasmin in extrahepatic bile duct cancer with advanced T stage and perineural invasion, suggesting a correlation with the severity of bile duct obstruction.

There are several limitations in this study. First, we did not measure the serum level of ceruloplasmin, because our study was retrospective. Second, we did not study the contribution of copper metabolism to cancer development and its progression, which needs to be evaluated in future prospective studies. In addition, the expression rate of ceruloplasmin in biliary epithelium had not previously been documented. In this study, the overall expression rate was 25.4% and only 5.1% showed strong expression, which left a relatively small number of patients for analysis. Furthermore, there was limited number of patient with ceruloplasmin strong expression without jaundice, therefore further analysis concerning the potential prognostic value of ceruloplasmin in relation with biliary obstruction was not conducted. Although we found a tendency for increased ceruloplasmin expression in advanced T stage cancer with perineural invasion, that finding did not achieve statistical significance. For the last, having no significant DEG for lymph node metastasis after age and sex adjustment, we included 157 genes with unadjusted p-value <0.05 in both T, N stage and perineural invasion. It would be more valuable to include DEGs with adjusted p-value <0.05, however, the shortage of sample inevitably lead to statistical limitation. A larger number of patients will be needed to validate our result in this study.

## MATERIALS AND METHODS

### Patients

Quality assessment of RNA for our experiment was performed using fresh frozen tumor tissue samples from 176 consecutive patients with intra- and extra-hepatic bile duct adenocarcinomas, along with normal bile duct tissue from 48 patients with ampulla of Vater adenocarcinoma, all of whom underwent surgical treatment at Seoul National University Hospital between year 2003 ~ 2011. After quality control, 79 samples of intra- and extra-hepatic bile duct adenocarcinoma and 21 samples of normal bile duct tissue were included in our experiments.

### Tissue collection and RNA extraction

Immediately after tumor resection, 5 × 5 mm pieces of tumor tissue and normal bile duct tissue were fresh frozen and stored in −70°C liquid nitrogen. Routinely processed 4-um thick paraffin-embedded sections from the same lesion were stained with hematoxylin and eosin and examined histologically.

RNA was extracted from tumor and normal bile duct tissue using RNeasy® kits (QIAGEN Sciences, Germantown, PA, USA), according to the manufacturer's instructions. RNA concentrations were determined spectrophotometrically, and RNA purity and integrity were evaluated by calculating the 260/280, and 260/239 ratios and by electrophoresis on 1% agarose gels [47].

### Microarray

Total RNA was submitted to DNA Link (Seoul, Korea) for gene expression profiling using the Affymetrix GeneChip® Human Gene 2.0 ST Array (Affymetrix, Santa Clara, CA, USA). Synthesis and labeling of cDNA targets and hybridization of GeneChips were carried out. Images were scanned with an Affymetrix GeneChip® Scanner 3000 7G (Affymetrix, Santa Clara, CA, USA). The quality of hybridization and overall chip performance were monitored by visually inspecting both internal quality control checks and the raw scanned data. Raw data were extracted and normalized using the robust multi-array average algorithm with Affymetrix GCOS software.

### Selection of DEG

The log 2 ratios of the samples were compared using permutation t-tests at simulation numbers of 100,000, adjusted for age and sex. An adjusted p value with false discovery rate correction less than 0.05 was considered significant. To find the DEGs with a linear association with increasing T, N stage and perineural invasion, DEGs that had positive coefficient were selected for further analysis. To evaluate the clinical effect of ceruloplasmin expression, we compared clinicopathological characteristics with the χ^2^-test. A p value of less than 0.05 was considered significant. The Kaplan-Meier method was used to calculate survival rates for candidate genes, compared using the log-rank test. All statistical analyses were performed in the R environment (The R Foundation for Statistical Computing, Vienna, Austria) and IBM SPSS Statistics version 21.0 (IBM Corp., Somers, NY, USA), with a p value less than 0.05 considered significant.

### Selection of candidate genes

We drew the candidate gene list by filtering for those with a positive log2 ratio in cancer tissue compared with normal bile duct tissue, using covariates of T, N stage and perineural invasion, adjusted for age and sex. Genes considered significant were assessed by gene functional classification, gene function and pathway annotation, and data mining to explore the association of each gene with disease. Gene function was annotated using the PANTHER database (http://www.pantherdb.org). Data mining to explore the relationship of each gene with disease was performed using the i-hop (http://www.ihop-net.org), OMIM (http://www.ncbi.nlm.nih.gov/omim), and oncomine (https://www.oncomine.org/) databases.

### Expression analysis of selected genes

Paraffin-embedded tissue samples were stained immunohistochemically with antibodies to ceruloplasmin (ab48614, Abcam, Cambridge, MA, USA, 1:200) according to the manufacturer's instructions. Immunohistochemical staining results were reviewed by a pathologist specializing in biliary-pancreatic disease with more than 10 years of experience. The intensity of staining was evaluated as a score of 0, 1, or 2+ for no staining, weak staining, and strong staining, respectively.

## SUPPLEMENTARY MATERIALS FIGURES AND TABLES








