# Supplementary material for: Ceruloplasmin as a prognostic marker in patients with bile duct cancer
Source: Oncotarget. 2017 Mar 7;8(17):29028–37. doi: 10.18632/oncotarget.15995 (PMC5438709; doi:10.18632/oncotarget.15995)
Supplement: Supplementary file 1 [file oncotarget-08-29028-s001.pdf]

Ceruloplasmin as a prognostic marker in patients with bile duct cancer

SUPPLEMENTARY TABLES

Supplementary Table 1: Top 50 genes with positive coefficient toward advanced T stage.

See Supplementary File 1

Supplementary Table 2: Top 50 genes with positive coefficient toward advanced N stage.

See Supplementary File 2

Supplementary Table 3: Top 50 genes with positive coefficient toward perineural invasion.

See Supplementary File 3
