# Supplementary material for: Ceruloplasmin as a prognostic marker in patients with bile duct cancer
Source: Oncotarget. 2017 Mar 7;8(17):29028–37. doi: 10.18632/oncotarget.15995 (PMC5438709; doi:10.18632/oncotarget.15995)
Supplement: Supplementary file 2 [file oncotarget-08-29028-s002.docx]

Supplementary table 1. Top 50 genes with positive coefficient toward advanced T stage

| Gene symbol | log2ratio | Fold change | p-value | Adjusted p-value |
| --- | --- | --- | --- | --- |
| SRGAP2B | 0.940284523 | 1.918906641 | 2.11E-08 | 0.001132 |
| NAP1L1 | 0.933499477 | 1.909903143 | 2.31E-07 | 0.003101 |
| DCBLD2 | 1.92631623 | 3.800834559 | 6.10E-07 | 0.004509 |
| RBMS1 | 1.160605654 | 2.235512564 | 6.81E-07 | 0.004509 |
| EFEMP1 | 1.859887323 | 3.629793117 | 9.65E-07 | 0.004637 |
| SRGAP2 | 0.832188952 | 1.780384631 | 1.39E-06 | 0.004986 |
| FAM171B | 1.386813738 | 2.615005061 | 3.37E-06 | 0.006701 |
| CNN3 | 1.112017389 | 2.161476857 | 3.06E-06 | 0.006701 |
| --- | 0.924960813 | 1.89863267 | 3.05E-06 | 0.006701 |
| LARP6 | 0.760174481 | 1.69369545 | 2.22E-06 | 0.006701 |
| SRGAP2B | 0.65798624 | 1.577878632 | 2.48E-06 | 0.006701 |
| LTBP3 | 0.742523986 | 1.673100358 | 3.63E-06 | 0.006704 |
| PKD2 | 0.8131433 | 1.757035451 | 4.27E-06 | 0.006937 |
| RAB12 | 0.576905373 | 1.491646185 | 5.55E-06 | 0.008049 |
| PTPN13 | 1.507718249 | 2.843599427 | 7.63E-06 | 0.009979 |
| FN1 | 1.701661579 | 3.252753687 | 1.22E-05 | 0.013637 |
| SRPX2 | 1.597427537 | 3.026032615 | 1.29E-05 | 0.014086 |
| CD109 | 1.594121697 | 3.019106599 | 1.60E-05 | 0.0148 |
| MYH10 | 1.243827626 | 2.368260246 | 1.52E-05 | 0.0148 |
| AKT3 | 1.080315152 | 2.114497935 | 1.68E-05 | 0.0148 |
| NUAK1 | 0.851767857 | 1.804711037 | 1.47E-05 | 0.0148 |
| FAM65A | 0.62555532 | 1.542804565 | 1.59E-05 | 0.0148 |
| ASPN | 1.701336305 | 3.252020395 | 2.52E-05 | 0.019183 |
| FLNA | 1.105844582 | 2.152248381 | 2.58E-05 | 0.019183 |
| SEMA3A | 1.175272386 | 2.258355154 | 2.89E-05 | 0.019585 |
| PTPRU | 0.952535143 | 1.935270381 | 3.79E-05 | 0.023365 |
| C1R | 1.135868811 | 2.197508599 | 3.84E-05 | 0.023367 |
| VCAN | 1.834397635 | 3.56622477 | 4.15E-05 | 0.02411 |
| ZNF772 | 0.598084608 | 1.513705563 | 4.11E-05 | 0.02411 |
| HDGFRP3 | 1.164942521 | 2.24224282 | 4.66E-05 | 0.025748 |
| PLS3 | 0.760929042 | 1.694581521 | 5.36E-05 | 0.028321 |
| CLIP4 | 0.933188773 | 1.909491863 | 6.14E-05 | 0.030486 |
| --- | 1.58926101 | 3.008951828 | 6.48E-05 | 0.030767 |
| EVC | 0.863234233 | 1.819111827 | 6.45E-05 | 0.030767 |
| PRELP | 1.724280563 | 3.30415316 | 6.88E-05 | 0.030826 |
| --- | 0.73374473 | 1.662949929 | 7.18E-05 | 0.030919 |
| SLC25A12 | 0.680455719 | 1.602645919 | 7.23E-05 | 0.030919 |
| SNORD114-22 | 1.591836199 | 3.014327558 | 7.80E-05 | 0.031497 |
| RDX | 0.725045597 | 1.652952877 | 7.78E-05 | 0.031497 |
| DZIP1 | 0.815390173 | 1.759774013 | 8.13E-05 | 0.031796 |
| TMEM136 | 0.575021244 | 1.489699399 | 8.56E-05 | 0.031885 |
| --- | 0.280073477 | 1.214256725 | 8.78E-05 | 0.032233 |
| ARHGEF25 | 0.758426734 | 1.691644872 | 9.13E-05 | 0.032865 |
| SNORD114-21 | 1.558292123 | 2.94504999 | 9.34E-05 | 0.03339 |
| HTRA1 | 1.066640892 | 2.094550825 | 9.57E-05 | 0.033769 |
| SLC39A10 | 0.565244679 | 1.479638433 | 9.95E-05 | 0.034203 |
| FGF2 | 1.477156684 | 2.783995122 | 0.000102 | 0.034286 |
| LINC00340 | 1.269363005 | 2.410551087 | 0.000101 | 0.034286 |
| GSTT2 | 0.715151764 | 1.641655902 | 0.000102 | 0.034286 |
| C1S | 1.327658735 | 2.509950192 | 0.000104 | 0.034663 |
