# Supplementary material for: Ceruloplasmin as a prognostic marker in patients with bile duct cancer
Source: Oncotarget. 2017 Mar 7;8(17):29028–37. doi: 10.18632/oncotarget.15995 (PMC5438709; doi:10.18632/oncotarget.15995)
Supplement: Supplementary file 3 [file oncotarget-08-29028-s003.docx]

Supplementary table 2. Top 50 genes with positive coefficient toward advanced N stage

| Gene symbol | log2ratio | Fold change | p-value | Adjusted p-value |
| --- | --- | --- | --- | --- |
| --- | 0.183349898 | 1.135517466 | 1.60E-05 | 0.640415 |
| --- | 0.784880819 | 1.72294998 | 0.00013 | 0.730975 |
| WDR96 | 0.648698529 | 1.56775327 | 0.00027 | 0.903145 |
| CYP24A1 | 1.272277802 | 2.415426248 | 0.000956 | 0.93037 |
| RIMKLB | 1.010151952 | 2.014123226 | 0.000433 | 0.93037 |
| SLC44A5 | 1.007876244 | 2.010948651 | 0.00101 | 0.93037 |
| ANXA8L1 | 0.948583195 | 1.929976385 | 0.00088 | 0.93037 |
| ANXA8 | 0.910845838 | 1.880147487 | 0.000779 | 0.93037 |
| FOXJ1 | 0.891679109 | 1.855334235 | 0.000894 | 0.93037 |
| ARMC3 | 0.719673258 | 1.646809023 | 0.000724 | 0.93037 |
| --- | 0.547389473 | 1.461438858 | 0.000773 | 0.93037 |
| CASC1 | 0.541161981 | 1.455144054 | 0.000758 | 0.93037 |
| --- | 0.411491185 | 1.330059868 | 0.000989 | 0.93037 |
| --- | 0.405784423 | 1.32480904 | 0.000555 | 0.93037 |
| CELSR2 | 0.404385993 | 1.323525501 | 0.001041 | 0.93037 |
| MDH1B | 0.366512396 | 1.28923244 | 0.001003 | 0.93037 |
| --- | 0.321318829 | 1.249472223 | 0.000613 | 0.93037 |
| CYP4B1 | 0.300115377 | 1.231242876 | 0.000331 | 0.93037 |
| --- | 0.254268854 | 1.192731124 | 0.00083 | 0.93037 |
| --- | 0.170890022 | 1.125752765 | 0.000993 | 0.93037 |
| --- | 0.115411569 | 1.083284039 | 0.001019 | 0.93037 |
| SRGAP3 | 0.362308441 | 1.285481138 | 0.001065 | 0.93634 |
| TTC18 | 0.442838475 | 1.359276048 | 0.001095 | 0.946549 |
| WDR65 | 0.179879529 | 1.132789289 | 0.00112 | 0.953171 |
| DSC3 | 0.98387549 | 1.977771137 | 0.001183 | 0.954315 |
| C1orf192 | 0.908921285 | 1.877641046 | 0.001572 | 0.954315 |
| ASB4 | 0.771418865 | 1.706947711 | 0.001204 | 0.954315 |
| ANXA8L2 | 0.702989327 | 1.627874327 | 0.00119 | 0.954315 |
| AQP3 | 0.646490047 | 1.565355184 | 0.001154 | 0.954315 |
| RNY4P19 | 0.628924691 | 1.54641195 | 0.001423 | 0.954315 |
| CLDN16 | 0.351221361 | 1.275640106 | 0.001562 | 0.954315 |
| MORN4 | 0.350648466 | 1.275133649 | 0.001339 | 0.954315 |
| --- | 0.340396266 | 1.266104308 | 0.001637 | 0.954315 |
| DNAH2 | 0.330444152 | 1.257400422 | 0.001579 | 0.954315 |
| KRT7 | 0.324459506 | 1.252195226 | 0.001307 | 0.954315 |
| --- | 0.30095938 | 1.231963386 | 0.00162 | 0.954315 |
| KRT6C | 0.250863079 | 1.18991876 | 0.001213 | 0.954315 |
| FSIP1 | 0.230819092 | 1.173501017 | 0.001454 | 0.954315 |
| AK8 | 0.229486703 | 1.17241774 | 0.00159 | 0.954315 |
| --- | 0.211519218 | 1.157906866 | 0.001593 | 0.954315 |
| --- | 0.19341371 | 1.143466191 | 0.001457 | 0.954315 |
| --- | 0.14078307 | 1.102503373 | 0.001468 | 0.954315 |
| --- | 0.082144498 | 1.058590417 | 0.001631 | 0.954315 |
| --- | 0.378474291 | 1.299966361 | 0.001675 | 0.965806 |
| --- | 0.523801147 | 1.437738349 | 0.001873 | 0.978482 |
| FLJ39051 | 0.211059435 | 1.157537904 | 0.001779 | 0.978482 |
|  | 0.531267387 | 1.445198224 | 0.002288 | 0.993489 |
| --- | 0.272558722 | 1.207948311 | 0.002138 | 0.993489 |
| RAET1E | 0.217571569 | 1.162774686 | 0.002037 | 0.993489 |
| --- | 0.209196774 | 1.156044373 | 0.002102 | 0.993489 |
