# Supplementary material for: Ceruloplasmin as a prognostic marker in patients with bile duct cancer
Source: Oncotarget. 2017 Mar 7;8(17):29028–37. doi: 10.18632/oncotarget.15995 (PMC5438709; doi:10.18632/oncotarget.15995)
Supplement: Supplementary file 4 [file oncotarget-08-29028-s004.docx]

Supplementary table 3. Top 50 genes with positive coefficient toward perineural invasion

| Gene symbol | log2ratio | Fold change | p-value | Adjusted p-value |
| --- | --- | --- | --- | --- |
| SEMA3A | 1.162049864 | 2.237751544 | 1.87E-06 | 0.027623 |
| --- | 1.149791365 | 2.218818048 | 1.15E-06 | 0.027623 |
| SRGAP2B | 0.720672958 | 1.647950557 | 2.06E-06 | 0.027623 |
| --- | 1.002823692 | 2.003918301 | 2.65E-06 | 0.028397 |
| --- | 0.987131951 | 1.982240416 | 5.09E-06 | 0.045441 |
| RARRES1 | 1.283643284 | 2.434530009 | 8.22E-06 | 0.055123 |
| MYH10 | 1.069307439 | 2.098425783 | 1.01E-05 | 0.059979 |
| PTGS2 | 1.471687215 | 2.77346057 | 3.17E-05 | 0.071245 |
| FGF2 | 1.336754926 | 2.52582542 | 2.92E-05 | 0.071245 |
| ANXA1 | 1.188891534 | 2.279775139 | 2.52E-05 | 0.071245 |
| FAM171B | 1.11876542 | 2.171610583 | 3.06E-05 | 0.071245 |
| PLAT | 1.108201069 | 2.15576672 | 1.49E-05 | 0.071245 |
| SESN3 | 0.886054251 | 1.84811464 | 3.19E-05 | 0.071245 |
| MEIS1 | 0.716136492 | 1.642776815 | 2.57E-05 | 0.071245 |
| SRGAP2 | 0.667674999 | 1.588510911 | 1.48E-05 | 0.071245 |
| IKZF2 | 0.66131278 | 1.581521074 | 3.18E-05 | 0.071245 |
| --- | 0.650039727 | 1.569211406 | 2.44E-05 | 0.071245 |
| LOC100131541 | 0.61351323 | 1.529980463 | 3.17E-05 | 0.071245 |
| ENDOD1 | 0.515369519 | 1.429360196 | 2.95E-05 | 0.071245 |
| TPM2 | 0.544254765 | 1.458266871 | 3.64E-05 | 0.078003 |
| ARHGAP23 | 0.645569345 | 1.564356522 | 4.27E-05 | 0.08475 |
| --- | 0.651543849 | 1.570848285 | 5.33E-05 | 0.097298 |
| MAML2 | 0.594577216 | 1.510030004 | 5.63E-05 | 0.097298 |
| GLI2 | 0.557871571 | 1.47209581 | 5.59E-05 | 0.097298 |
| MXRA5 | 1.417470126 | 2.671166909 | 6.38E-05 | 0.101654 |
| TACSTD2 | 1.286901699 | 2.440034758 | 6.83E-05 | 0.101654 |
| CRABP2 | 0.796414922 | 1.736779885 | 6.63E-05 | 0.101654 |
| DUSP7 | 0.62551085 | 1.54275701 | 6.82E-05 | 0.101654 |
| DPYSL3 | 1.10409619 | 2.149641666 | 7.22E-05 | 0.101838 |
| ZNF462 | 0.716175024 | 1.642820692 | 7.15E-05 | 0.101838 |
| CMTM7 | 0.69211807 | 1.615653775 | 7.50E-05 | 0.10315 |
| VCAN | 1.612904184 | 3.05866941 | 8.44E-05 | 0.105267 |
| TWSG1 | 0.675801702 | 1.597484244 | 8.05E-05 | 0.105267 |
| PDE3A | 1.087475418 | 2.125018525 | 0.000103 | 0.108302 |
| FSTL1 | 0.990126539 | 1.986359206 | 0.000102 | 0.108302 |
| FOXC1 | 0.869014686 | 1.826415092 | 9.77E-05 | 0.108302 |
| RBMS1 | 0.856906115 | 1.8111501 | 9.80E-05 | 0.108302 |
| --- | 0.446597033 | 1.362821897 | 9.67E-05 | 0.108302 |
| GLI3 | 0.759336835 | 1.692712356 | 0.000109 | 0.112903 |
| SFRP2 | 2.702398539 | 6.508831378 | 0.000137 | 0.124336 |
| CACNG4 | 1.271430559 | 2.414008171 | 0.000143 | 0.124336 |
| BMPR1B | 0.956194611 | 1.940185523 | 0.000137 | 0.124336 |
| NXN | 0.883079349 | 1.844307671 | 0.000137 | 0.124336 |
| NRP2 | 0.883060726 | 1.844283864 | 0.000131 | 0.124336 |
| EHD2 | 0.819459366 | 1.76474455 | 0.000128 | 0.124336 |
| --- | 0.738111829 | 1.667991375 | 0.00013 | 0.124336 |
| LBH | 0.486913003 | 1.401442938 | 0.000141 | 0.124336 |
| ANTXR1 | 1.057033694 | 2.080649126 | 0.000147 | 0.124839 |
| PRDM6 | 0.587264014 | 1.502394841 | 0.000149 | 0.124839 |
| BAG2 | 0.695555291 | 1.619507659 | 0.000165 | 0.125452 |
